# The Combined Influences of Exercise, Diet and Sleep on Neuroplasticity

**DOI:** 10.3389/fpsyg.2022.831819

**Published:** 2022-04-26

**Authors:** Jacob W. Pickersgill, Claudia V. Turco, Karishma Ramdeo, Ravjot S. Rehsi, Stevie D. Foglia, Aimee J. Nelson

**Affiliations:** ^1^Department of Kinesiology, McMaster University, Hamilton, ON, Canada; ^2^Faculty of Medicine and Dentistry, University of Alberta, Edmonton, AB, Canada; ^3^School of Biomedical Engineering, McMaster University, Hamilton, ON, Canada

**Keywords:** exercise, diet, sleep, neuroplasticity, aerobic, resistance, lifestyle

## Abstract

Neuroplasticity refers to the brain’s ability to undergo structural and functional adaptations in response to experience, and this process is associated with learning, memory and improvements in cognitive function. The brain’s propensity for neuroplasticity is influenced by lifestyle factors including exercise, diet and sleep. This review gathers evidence from molecular, systems and behavioral neuroscience to explain how these three key lifestyle factors influence neuroplasticity alone and in combination with one another. This review collected results from human studies as well as animal models. This information will have implications for research, educational, fitness and neurorehabilitation settings.

## Introduction

Contrary to prior assumptions regarding the rigidity of the mammalian nervous system during adulthood, mounting neuroscientific evidence has acknowledged that the adult brain changes in response to our experiences. Neuroplasticity refers to the brain’s ability to undergo structural and functional reorganization in response to learning or experience. The classic theory which posits that “neurons that fire together, wire together” (Hebb, 1949) accurately describes how plasticity of the brain is the result of strengthening the connections between neurons by repetitive firing patterns which activates distinct neurobiological mechanisms. Conversely, the “use it or lose it” principle also applies to neuroplasticity, where a lack of activity between neurons within a circuit leads to a decrease in the strength of connection between these neurons (Liepert et al., 1995). The strengthening of synaptic connections between neurons is known as long-term potentiation (LTP), and the activity-dependent reduction in strength is known as long-term depression (LTD) (Bear and Malenka, 1994). These processes are thought to be mediated by the activity of N-methyl-D-aspartate (NDMA) glutamate receptors (Peters et al., 2018). However, neuroplastic adaptations can be evaluated in humans using several neuroscientific techniques since changes occur at the molecular, cellular, systems, and behavioral levels (El-Sayes et al., 2019a). Each of these forms of measures are reflective of different aspects of neural activity, and neuroplasticity refers to any changes in the structure or function of our brain activity. Therefore, from this point forward in the review, neuroplasticity will be defined as changes in any of these measures discussed.

The timeline of neurophysiological processes underlying neuroplasticity varies from rapid adaptations of synaptic strength that are transient in nature, to more long-term structural modifications (Sagi et al., 2012). Molecular mechanisms including synaptogenesis (formation of new synaptic connections), neurogenesis (development of new neurons), angiogenesis (formation of new blood vessels) and gliogenesis (generation of non-neuronal glial cells in the brain) contribute to neuroplasticity (El-Sayes et al., 2019a). Cellular adaptations in response to experience include presynaptic changes such as greater neurotransmitter and neurotrophin release from the presynaptic neuron, decreased neurotransmitter reuptake and breakdown in the synaptic cleft, and postsynaptic adaptations resulting in deposition of additional neurotransmitter receptors on the cell membrane (Gulyaeva, 2017). From a systems neuroscience perspective, the size of the neural representation of muscles in the motor cortex increases in response to frequent and repetitive use of the muscle (Pascual-Leone et al., 1993). The increased cortical representation means that more neurons in the brain are dedicated to controlling that muscle. Increased cortical representation facilitates improved motor control of those muscles, revealing dynamic functional neuroplasticity as a result of experience (Martin, 2005). Conversely, neuroplasticity can also occur in the brain when intact adjacent brain regions overtake the function of nearby neurons disrupted by brain injury or disease (Leemhuis et al., 2019). Behavioral approaches to studying neuroplasticity also highlight how changes in the structure and function of neural circuits are associated with learning (Sagi et al., 2012), memory (Amtul and Atta-Ur-Rahman, 2015), and cognitive function (Vance et al., 2010). Changes in cognitive functioning serve as a measure of neuroplasticity because the improvements in performance on a cognitive task represent changes in the activity of the neural networks which execute these mental processes (Ji et al., 2017).

**FIGURE 1:**
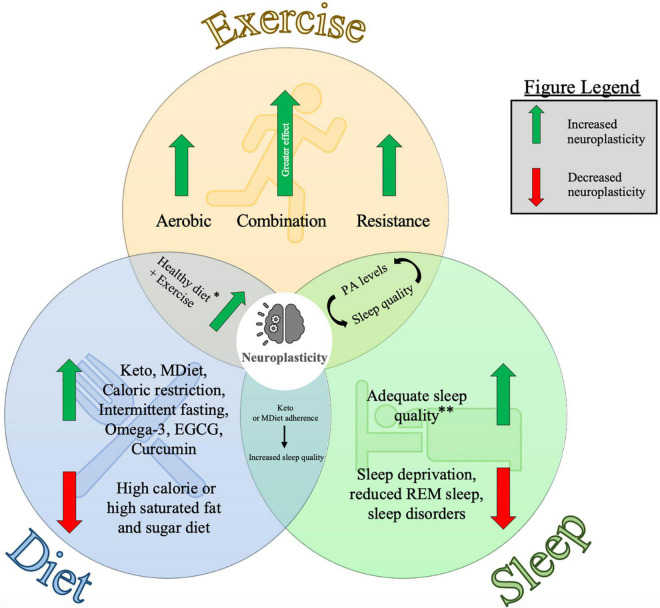
Diagram representing the individual and combined influences of exercise, diet and sleep on neuroplasticity. This figure summarizes the key points from each of the major sections of this article and visually depicts how they are interrelated. Findings from this review are from studies in human and animal models. Neuroplasticity was measured using several techniques including neurophysiological measures, molecular biomarkers, and cognitive assessments (see Tables 1, 2 for further information). Keto: Ketogenic diet, MDiet: Mediterranean diet, EGCG: (–)epigallocatechin-3-gallate, REM sleep: rapid-eye movement sleep, PA levels = physical activity levels. ^∗^Healthy diet here refers to evidence from the Ketogenic diet, Mediterranean diet and nutritional supplements. ^∗∗^Adequate sleep quality is specific to individuals depending on age and other health factors. See Ohayon et al. (2017) for further information related to sleep recommendations.

The magnitude of neuroplasticity that occurs in response to learning or experience is partially dependent on factors that influence brain activity and the predisposition for plasticity induction. Exercise, diet, and sleep are three behaviours that represent essential pillars of mental health because of their impact on the structure and function of the brain (Wickham et al., 2020). It is well understood that good nutrition, regular exercise and sufficient sleep are fundamental to maintaining a healthy lifestyle (Farooqui, 2014). A cross-sectional study revealed that sleep quality, adequate fruit and vegetable intake, and regular physical activity improve depressive symptoms and wellbeing in young adults (Wickham et al., 2020). However, the role of exercise, diet, sleep, and the combined influence of these factors on neuroplasticity is not yet fully understood. The purpose of this review is to discuss evidence from human and animal models that demonstrates the role of these three fundamental lifestyle factors in facilitating neuroplasticity from a behavioral, molecular, and systems neuroscience perspective. A stronger understanding of these influences will allow students and educators to better understand how exercising, eating healthy and getting proper sleep can benefit learning and memory for educational purposes. Findings from this review will also permit neurophysiology researchers to recognize how these factors influence neurophysiological biomarkers of neuroplasticity and provide them with suggestions for potential methods to account for these factors. Further, this review will help identify the importance of considering these factors when attempting to maximize exercise-induced neuroplasticity. As such, findings from this review have implications for educational, research, fitness and neurorehabilitation settings. A graphical summary of the main findings from each of the major sections in this review can be found in Figure 1.

**TABLE 1:** Neurophysiological and molecular measures of neuroplasticity.

*Measures*	*Method*	*Protocol*	*Interpretation*
*MEP*	TMS	Single-pulse TMS over M1	Corticospinal excitability
*RMT*	TMS	Single-pulse TMS over M1	Minimum stimulation intensity required to evoke an MEP
*CSP*	TMS	Single-pulse TMS over M1 during muscle activity	Intracortical and spinal excitability suppression of the contralateral target muscle
*SICI*	TMS	Paired-pulse TMS over M1: a subthreshold stimulus in advance (1–6 ms) of a suprathreshold stimulus	Cortical suppression of MEP amplitude
*LICI*	TMS	Paired-pulse TMS over M1: a suprathreshold stimulus followed (50–200 ms) by a suprathreshold stimulus	Cortical suppression of MEP amplitude
*ICF*	TMS	Paired-pulse TMS over M1: a subthreshold stimulus in advance of a subthreshold stimulus (8–30 ms)	Cortical facilitation of MEP amplitude
*SAI*	TMS and PNS	Single-pulse TMS over M1 preceded by PNS stimulation (20–25 ms)	Cortical suppression of MEP amplitude reflecting sensorimotor integration
*rTMS*	TMS	Rapid (5–10 Hz) succession of pulses	Plasticity-inducing TMS paradigm leading to lasting increases in corticospinal excitability
*cTBS*	TMS	Clusters of three TMS pulses delivered in rapid succession	Plasticity-inducing rTMS paradigm resulting in LTD and cortical excitability suppression
*PAS*	TMS and PNS	Repeated pairings of TMS and PNS pulses delivered in rapid succession (50–150 pairings)	Plasticity-inducing paradigm to strengthen corticospinal excitability
*ERP*	EEG	Latency and amplitude of cortical activity evoked by exposure to repeated stimuli such as lights or audible tones	Sensory processing and cognitive functioning
*Cerebral blood flow*	TCD Ultrasound	Recorded from cerebral arteries to measure rate and velocity of blood flow in the brain	Hemodynamic activity which facilitates neurovascular coupling and brain function
*BDNF*	Blood draw	Blood serum or plasma levels measured using ELISA	Concentration of circulating BDNF which promotes neuroplasticity and memory
*IGF-1*	Blood draw	Blood serum or plasma levels measured using ELISA	Concentration of circulating IGF-1 which promotes brain development and maintenance
*VEGF*	Blood draw	Blood serum or plasma levels measured using ELISA	Concentration of circulating VEGF which promotes neural growth and proliferation

*BDNF, brain-derived neurotrophic factor; CSP, contralateral silent period; cTBS, continuous theta-burst stimulation; EEG, electroencephalography; ELISA, enzyme-linked immunosorbent assay; ERP, event-related potential; ICF, intracortical facilitation; IGF-1, insulin-like growth factor 1; LICI: long-interval intracortical inhibition; M1, primary motor cortex; MEP, motor-evoked potential; PAS, paired-associative stimulation; PNS, peripheral electrical nerve stimulation; RMT, resting motor threshold; rTMS, repetitive TMS; SAI, short-latency afferent inhibition; SICI, short-interval intracortical inhibition; TCD, transcranial doppler; VEGF, vascular endothelial growth factor.*

**TABLE 2:** Cognitive assessments of neuroplasticity.

*Measure*	*Protocol*	*Interpretation*
*Trail making test*	Pencil used to connect a series of 25 encircled numbers and letters in numerical and alphabetical order	Visual attention and task-switching
*Stroop test*	Series of color words presented in an incongruent color; respond with the color of the word, not what the word says	Response inhibition
*Flanker test*	Series of arrows pointing in different directions presented; respond with the direction of central arrow in the series	Attention and executive function
*Digit-span backward*	Pairs of random number sequences read; respond with the sequence in reverse order	Working memory
*Fugl-meyer score*	Movements are observed and measured on an ordinal scale; assessment of motor functioning, balance, joint pain and ROM	Voluntary motor control of upper and lower extremities
*SIS*	64 item self-report measure of strength, hand function, ADLs, mobility, communication, emotion, memory/thinking and participation	Emotional, social and cognitive functions
*MMSE*	30 item questionnaire measuring orientation, registration, attention/calculation, recall, and language	Cognitive impairment
*MoCA*	30 point self-report measure of visuospatial skills, executive function, naming, attention/concentration, serial subtraction, language, abstraction, delayed recall, and orientation	Cognitive dysfunction

*ADL, activities of daily living; MMSE, mini mental status examination; MoCA: Montreal Cognitive Assessment; ROM: range of motion; SIS, stroke index scale.*

## Exercise

### Aerobic Exercise

Aerobic exercise refers to physical activities such as biking, running, or swimming that are repetitive in nature and promote the circulation of oxygen through the cardiovascular system. Aerobic exercise enhances the expression of neuroplasticity biomarkers including brain-derived neurotrophic factor (BDNF), insulin-like growth factor 1 (IGF-1), and vascular endothelial growth factor (VEGF) (Cotman et al., 2007; Knaepen et al., 2010; Mang et al., 2013). The cascade of molecular changes upregulates cellular processes of synaptogenesis, neurogenesis, angiogenesis and gliogenesis (El-Sayes et al., 2019a), elevates cerebral blood flow (Smith J. C. et al., 2010; Zimmerman et al., 2014), increases grey and white matter volume (Colcombe et al., 2006; Erickson et al., 2014; Fletcher et al., 2016; Sexton et al., 2016), and neuronal activity (Moraes et al., 2007; Bailey et al., 2008; Rajab et al., 2014; Gutmann et al., 2015). As such, aerobic exercise has been linked to improved cognitive and motor function. In a group of 10 young healthy individuals, 30 min of moderate-intensity cycling improved performance of several measures of memory, reasoning, concentration and planning compared to baseline (Nanda et al., 2013). In another study, 24 healthy young participants performed a pinch-grip motor task before and after 30 min of moderate intensity running. Results indicated that an acute bout of aerobic exercise led to improved motor skill acquisition mainly driven by improvements in accuracy (Statton et al., 2015). These findings demonstrate the usefulness of aerobic exercise as an adjunct tool for rehabilitation, as it appears to prime the nervous system for learning and skill acquisition. The effectiveness of exercise to promote cognitive functioning and neuroplasticity has been demonstrated in young and older healthy individuals (Sibley et al., 2006; Wang et al., 2016), as well as clinical populations such as stroke (Moriya et al., 2016), multiple sclerosis (Sandroff et al., 2015) and depression (Vasques et al., 2011). Aerobic exercise also aided in functional recovery and learning by mobilizing neural resources which prepare the brain for subsequent performance on cognitive tasks (Moriarty et al., 2019).

The capacity for aerobic exercise to induce neuroplasticity can be assessed using transcranial magnetic stimulation (TMS). TMS is a non-invasive form of brain stimulation that provides a measurement of cortical and corticospinal excitability. A change in corticospinal excitability following an acute bout of exercise is suggested to indicate that neuroplasticity has been induced. As reviewed previously (Turco and Nelson, 2021), studies have reported inconsistent changes in TMS measures following aerobic exercise. As an example, a single session of aerobic exercise has been reported to increase (Lulic et al., 2017; Neva et al., 2017; El-Sayes et al., 2019b; MacDonald et al., 2019; Nicolini et al., 2020) or not change (McDonnell et al., 2013; Singh et al., 2016; Brown et al., 2020; El-Sayes et al., 2020) corticospinal excitability as assessed with motor-evoked potentials (MEPs). Further, short-interval intracortical inhibition (SICI), an assessment of primary motor cortex (M1) microcircuitry, has been reported to decrease (Singh et al., 2014; Smith et al., 2014; Stavrinos and Coxon, 2017; Yamazaki et al., 2019) or not change (Mooney et al., 2016; Andrews et al., 2020; Morris et al., 2020; Hu et al., 2021) following a single session of aerobic exercise. Studies have also shown inconsistent effects of aerobic exercise on other common TMS measures including the contralateral silent period (CSP), intracortical facilitation (ICF), long-interval intracortical inhibition (LICI), and short-latency afferent inhibition (SAI) (see Turco and Nelson, 2021). Table 1 provides descriptions of the neurophysiological and molecular measures of neuroplasticity that are discussed in this review. Future research should continue to explore the effect of aerobic exercise on these TMS measures, since the conflicting results reported here could be due to differences in the type, intensity or volume of aerobic exercise prescribed, as well as the fitness levels of the individuals in the study.

### Resistance Exercise

Resistance exercise refers to repeated contraction of muscles against an external resistance. Some examples of resistance exercises include bodyweight exercises like pushups, and the use of dumbbells, resistance bands or exercise machines. This form of training leads to an increase in muscle strength driven by cellular responses in the short-term (days-weeks) that transition to structural changes through synaptogenesis following long-term training (months-years) (Tallent et al., 2021). A meta-analysis of 31 studies showed that resistance training increases MEPs and reduces SICI and CSP when the muscle is active (Siddique et al., 2020). This suggests that short-term resistance training overall leads to an increase in corticospinal output to the trained muscle. Evidence showing that resistance training leads to enhanced corticospinal output to the trained as well as the untrained limb demonstrates the global impact of training-induced neuroplasticity. For instance, MEP amplitude increased and SICI decreased in the untrained leg following 9 training sessions of single right leg squatting (Goodwill et al., 2012). No studies have been conducted to measure corticospinal adaptations to resistance training performed greater than 3 months in duration. Therefore, research on long-term adaptations is limited to findings from studies using a cross-sectional design. Studies on individuals with multiple years of resistance training experience found no differences in corticospinal excitability when compared to untrained controls, despite differences in strength (Del Olmo et al., 2006; Tallent et al., 2013). Another study reported a significant increase in spinal excitability with no differences in corticospinal excitability compared to controls in the biceps brachii of the non-dominant arm (Philpott et al., 2015). Future work should focus on implementing longitudinal designs to assess spinal and supraspinal adaptations following resistance training regimens lasting several months to several years in duration. Although these types of studies may pose some logistical challenges, they will provide fundamental new insights into the neural responses to chronic resistance training and allow us to begin to understand how they differ from acute and short-term responses.

Results from functional magnetic resonance imaging (fMRI) have showed greater hemodynamic activity in the anterior left middle temporal gyrus, left anterior insula and lateral orbital frontal cortex following 12 months of twice weekly resistance training in healthy senior women. These hemodynamic changes were also associated with improvements in cognition measured using the Flanker test (Liu-Ambrose et al., 2011). Performance improvements on the Flanker test induced by resistance training were partly mediated by more efficient engagement of response inhibition processes. It is also suggested that improvements in cognitive performance and neuroplasticity following training are influenced by mechanisms involving elevated IGF-1 (Cassilhas et al., 2007) and reduced homocysteine levels (Vincent et al., 2003). Another fMRI study investigated changes in functional connectivity and memory following resistance training in elderly women with Mild Cognitive Impairment. Results indicated that resistance training increased activity in three regions of the cortex during encoding and recall processes: right lingual gyrus, occipital fusiform gyrus, and the right frontal pole (Nagamatsu et al., 2012). There was also a positive correlation between the increased activity in right lingual gyrus and improvements in associative memory performance (Nagamatsu et al., 2012). These findings from fMRI studies further support the notion that exercising leads to increased activity in several brain regions implicated in important cognitive functions, and that these changes facilitate improved performance on assessments of these cognitive abilities.

Electroencephalography (EEG) is another neuroimaging method that has been used to investigate neural adaptations in response to resistance exercise. Event related potentials (ERP’s) are EEG measures that provide a non-invasive approach to assess brain functioning by measuring electrical activity over the scalp in response to repeated stimuli such as flashing lights or audible tones. The ERP is composed of several components identified by their polarity and order of occurrence. For example, the P1 component represents the first positive component of the response following stimulus onset, while N2 represents the second negative component (Beck and Dustman, 1975). Early ERP components reflect arrival and processing of sensory input, while later components are related to more complex cognitive functions such as attention and working memory (Kügler et al., 1993). In one study performed in older adults, participants were randomized into either a control, aerobic or resistance training group and underwent 9 weeks of training with three sessions per week. ERPs were collected in response to an auditory tone before and after training. Results from this study indicated that resistance training reduced latencies of the N1, N2, and P2 components as well as increased amplitudes of the N1P2, P2N2, and N2P3 components at the Cz and Fz locations (Özkaya et al., 2005). It was suggested that these changes in ERP’s highlight how resistance training can facilitate sensory processing and cognitive function in an older population (Özkaya et al., 2005).

### Combination of Aerobic and Resistance Exercise

Resistance exercise leads to neurophysiological adaptations through mechanisms unique from those induced by aerobic training (Cassilhas et al., 2012; Vilela et al., 2017). Evidence has suggested that major contributors to exercise induced neuroplasticity following both aerobic and resistance training are related to increased cerebral blood flow and increased IGF-1 levels (Loprinzi et al., 2020). However, aerobic exercise has a greater influence on BDNF levels, while resistance exercise is thought to have a greater influence on IGF-1 levels and reductions in Interleukin 6 (IL-6) levels (Loprinzi et al., 2020). Spatial memory is influenced by differential molecular pathways depending on the modality of exercise performed (Cassilhas et al., 2012). In a study using rats, it was found that aerobic training increased hippocampal levels of IGF-1, BDNF, tropomyosin receptor kinase B (TrkB), and calmodulin-dependent protein kinase II (β-CaMKII). Alternatively, resistance training increased the peripheral and hippocampal levels of IGF-1, along with activation of protein-kinase B (AKT) and the IGF-1 receptor (Cassilhas et al., 2012). In another study using rats, aerobic treadmill training was compared to ladder climbing with a weight attached to the rat’s tail (resistance training). Aerobic and resistance exercise both lead to improved spatial memory along with increased expression of proteins involved in synaptic plasticity (Vilela et al., 2017). However, each type of training resulted in unique molecular adaptations related to signaling proteins and neurotrophic factors. Specifically, aerobic exercise increased the expression of glutamatergic proteins and decreased DNA damage, while resistance training increased protein kinase C alpha (PKC α), tumor necrosis factor (TNF- α) and IL-1 β levels.

Due to these unique neurophysiological adaptations that occur in response to aerobic compared to resistance exercise discussed, studies have found that the combination of aerobic and resistance exercise can lead to greater levels of exercise-induced neuroplasticity than either type alone (Marinus et al., 2019). Meta-analyses have found that improvements in cognitive domains including attention, processing speed and working memory are greater following a combination of aerobic and resistance training compared to either form of training alone (Colcombe and Kramer, 2003; Smith P. J. et al., 2010). Many behavioral studies have also demonstrated improvements in cognition following aerobic and resistance training using several psychological tools which are described further in Table 2 (Cassilhas et al., 2007; Liu-Ambrose et al., 2010; Kluding et al., 2011; Nagamatsu et al., 2012; Shafer, 2020). Further, several studies have explored the influence of combined training on neurophysiological measures of neuroplasticity as well. For example, 6 months of performing a combined aerobic and resistance exercise training protocol significantly increased serum BDNF levels in healthy middle-aged women compared to a non-exercising control group (Cho et al., 2014). Combined training did not induce significantly larger changes in BDNF levels compared to aerobic training only in this study, but authors suggested that this may have been due to the low level of exercise intensity prescribed in this study (1 set of 30/40% 1RM) (Cho et al., 2014). However, another study that assessed changes in BDNF levels following either aerobic, resistance, or combined training in adolescents with obesity had different results. There were no significant increases in BDNF levels in the aerobic, resistance or combined groups compared to a diet-only control group following a 22-week training intervention (Goldfield et al., 2018). These conflicting findings regarding changes in BDNF may be limited to the specific age, sex or fitness levels of the individuals collected in these studies, as well as the differences in the specific frequency, intensity, time and type of aerobic and resistance exercise prescribed by the investigators. For this reason, further research in humans is needed to determine the relationship between combined exercise protocols and changes in BDNF and other biomarkers of neuroplasticity. This would improve our current understanding of the neurophysiological benefits of combining both types of training, as well as the optimal balance of the two types for individual fitness goals.

Improvements in cognition have also been reported following aerobic combined with resistance training in clinical populations. A randomized control trial in Chronic Obstructive Pulmonary Disease (COPD) reported additional improvements in multiple cognitive functions including long-term memory, apraxia, and reasoning skills compared to aerobic training only (Aquino et al., 2016). Similar findings were also reported in other reviews on the effects of fitness training on cognitive function in older adults (Colcombe and Kramer, 2003; Smith P. J. et al., 2010). The effects of combined exercise, aerobic exercise, or no exercise on cognitive function following 9 weeks of training was investigated in 109 patients with Dementia. The most pronounced improvements in cognitive (higher global cognition, visual memory, verbal memory and executive function) and motor (walking endurance, leg muscle strength and balance) performance occurred in the combined training group (Bossers et al., 2015). These findings demonstrate that combined training is more effective than aerobic training in isolation for slowing cognitive and motor decline in patients with dementia. However, improvements in motor and cognitive functioning returned toward baseline 9 weeks after training. This trend highlights the need for regular exercise to be able to acquire and maintain the neurological benefits of combined training. This is important information that can inform future studies trying to determine the minimum frequency and intensity of exercise required for patients with dementia, as well as other special populations.

Other studies in clinical populations have shown that exercise training led to improvements in other clinical cognitive assessments including the Fugl-Meyer Score and Stroke Impact Scale (SIS) (Kluding et al., 2011), Mini Mental Status Examination (MMSE) (Lee et al., 2016) and the Montreal Cognitive Assessment (MoCA) (Marzolini et al., 2013). These cognitive assessments of neuroplasticity are described further in Table 2. Overall, the evidence reviewed here suggests that aerobic and resistance exercise each profoundly influence neuroplasticity through distinct mechanisms. Further, it is likely that the combination of these types of training induces the greatest amount of exercise-induced neuroplasticity, both in healthy and clinical populations. This information may have considerable implications for neurorehabilitation programs that aim to recover motor and cognitive functioning in diseased states, or fitness programs aiming to enhance the mental and neurological benefits of exercise training. Nonetheless, further research in humans is still necessary to clarify the neurophysiological mechanisms underpinning these synergistic benefits in acute and prolonged exercise protocols.

## Diet

Diet is a lifestyle factor known to influence brain structure and function that could alter the propensity for neuroplasticity (Gomez-Pinilla and Gomez, 2011; Murphy et al., 2014). Diet influences TMS measures of cortical and corticospinal activity. Acute intake of glucose increases MEPs and LICI (Specterman et al., 2005), with no change in SICI, ICF or resting motor threshold (RMT) (Badawy et al., 2013). However, glucose intake does not always influence corticospinal excitability (Toepp et al., 2019). The ketogenic diet and the Mediterranean diet (high in vegetables, fruits, legumes, nuts, grains, fish, and unsaturated fats but low in meat and dairy) both claim to support brain function among several other benefits (Petersson and Philippou, 2016). The Mediterranean diet appears to facilitate these benefits by preserving the integrity and structural connectivity of essential brain circuits associated with cognitive functioning (Pelletier et al., 2015). It has also been shown that adhering to a ketogenic diet for 2 weeks led to an increase in SICI, with no change in RMT, CSP or ICF (Cantello et al., 2007). Alternatively, a gluten-free diet decreases RMT without changes in CSP, MEP’s, SICI or ICF in patients with Celiac Disease (Bella et al., 2015). Together, these results identify the influence of several dietary factors on TMS measures of cortical and corticospinal activity.

Dietary interventions such as caloric restriction, intermittent fasting, and diet supplementation modulate markers of neuroplasticity and influence cognition and mood (Murphy et al., 2014). For example, caloric restriction involves a 20–40% reduction in daily caloric intake (Lee et al., 2002). Caloric restriction elevates BDNF levels (Lee et al., 2002), enhances performance on memory tests in humans (Witte et al., 2009) and sensorimotor function and learning in rats (Singh et al., 2012). Further, caloric restriction has also been shown to have positive implications for clinical populations including reduced risk of developing Alzheimer’s Disease (Luchsinger et al., 2002) and reduced severity of neurochemical imbalances and motor dysfunction in a primate model of Parkinson’s Disease (Maswood et al., 2004). Intermittent fasting is a form of restrictive eating where individuals cycle through stages of voluntary fasting and non-fasting throughout a day. An intermittent fasting cycle of 16 h fasting and 8 h fed has also been shown to increase hippocampal neurogenesis in mice, and this relationship is thought to be mediated by mechanisms involving Notch 1 signaling (Baik et al., 2020).

There is increasing interest in the use of dietary interventions to improve symptoms in Autism (Cieślińska et al., 2017), Alzheimer’s Disease (Rusek et al., 2019) and Parkinson’s Disease (Shah and Duda, 2015). There are the positive effects of the ketogenetic diet on the brain and various clinical conditions. The ketogenic diet is a high-fat, adequate-protein, low-carbohydrate diet which shifts energy metabolism to rely on fats instead of carbohydrates and has been used as an intervention for individuals with epilepsy (D’Andrea Meira et al., 2019). The ketogenic diet has proposed neuroprotective effects including a reduction in free radicals (Gasior et al., 2006; Masino and Rho, 2012), upregulation of the glutathione antioxidant enzyme in the hippocampus (Ziegler et al., 2003), reduction in inflammation, and normalization of synapses between neurons (Stafstrom and Rho, 2012). In individuals with Alzheimer’s Disease, the ketogenic diet improved cognitive function (Henderson et al., 2009; Kashiwaya et al., 2013; Ota et al., 2018), motor function (Beckett et al., 2013; Brownlow et al., 2013) and reduced beta amyloids and tau proteins in the brain (Van DerAuwera et al., 2005; Studzinski et al., 2008). In Parkinson’s Disease, the ketogenic diet relieved motor and non-motor symptoms (Phillips et al., 2018), reduced cell death (Cheng et al., 2007) and improved motor performance (Tieu et al., 2003; Shaafi et al., 2016). However, neurological conditions like these have a complex pathophysiology that can negatively influence processes of neuroplasticity in the brain. To discuss these effects in detail is beyond the scope of this review. To learn more about this, see the following reviews on neuroplasticity in special populations including Alzheimer’s Disease (Koch and Spampinato, 2022), Parkinson’s Disease (Hirsch and Farley, 2009), Stroke (Hara, 2015) and other neurological conditions (Thickbroom and Mastaglia, 2009).

Supplementation of omega-3 fatty acids, catechin polyphenols and curcumin have been shown to support cognitive processes (McCann and Ames, 2005) and exert neuroprotective effects (Murphy et al., 2014). Potential mechanisms of the effect of omega-3 fatty acids on neuroplasticity include its influence on depolarization-induced glutamate transmitter release, increased hippocampal neurotrophin levels (Wu et al., 2004) and increased expression of genes essential for synaptic plasticity (Wu et al., 2007). Docosahexaenoic acid (DHA) is an omega-3 fatty acid that allows for phospholipid signal transduction to occur at the synapse. This is an essential process for brain glucose uptake and preventing oxidative stress (Pifferi et al., 2005). Mechanisms involved in learning, memory, and cognitive decline are modulated by catechin polyphenols (Mandel and Youdim, 2004; Vauzour, 2012). Their antioxidant properties allow them to release hydrogens to reduce the reactive oxygen species and free radicals in the body. (–)epigallocatechin-3-gallate (EGCG), a catechin polyphenol has been shown to be implicated in synaptic plasticity as well as learning and memory (Wroblewski and Danysz, 1989; Bliss and Collingridge, 1993; Haque et al., 2006). EGCG modulates glutamate release by upregulating calcium entry which in turn activates PKC, a pathway correlated to learning and memory (Levites et al., 2003; Chou et al., 2007). Studies have shown that the structure of curcumin may underly its positive effects on brain function, as it can move across the blood brain barrier (Garcia-Alloza et al., 2007) and reduces oxidative stress by transferring electrons to readily collect free radicals (Phillips et al., 2018). Curcumin supplementation for 3 weeks before and after induced traumatic brain injuries (Wu et al., 2006; Sharma et al., 2010) led to increased BDNF and cAMP response element binding proteins. These are both markers of synaptic plasticity and facilitation of neurogenesis of the hippocampus in rats (Wu et al., 2006). This supplement has also been found to modulate chronic inflammation via lowering TNF- alpha concentration levels which aid neurotransmission and neurogenesis (Sahebkar et al., 2016). Alternatively, there is evidence suggesting that unhealthy dietary habits such as caloric heavy diets and consumption of saturated fats and sugars can have negative consequences on brain health and functioning (Molteni et al., 2002; Gomez-Pinilla, 2008). Spatial learning and BDNF levels in the hippocampus were reduced in rats following 2 months on a high saturated fat and refined sugar diet. These findings were interpreted as impairments in synaptic plasticity and motor learning. In support of this, the chronic consumption of a diet high in saturated fat is associated with an increased risk of cognitive decline and eventually dementia through mechanisms mediated by the development of insulin resistance and type II diabetes (Greenwood and Winocur, 2005). Although the evidence of dietary interventions is promising, it is important to note that these studies have been done in animal models and future steps require more widespread evaluations in human populations.

In summary, an individual’s dietary choices clearly effect neuroplastic processes in the brain. Adherence to certain dietary interventions such as the Mediterranean diet, ketogenic diet, caloric restriction, intermittent fasting and diet supplementation appear to increase measures of neuroplasticity in the brain. Therefore, routine consumption of unhealthy diets such as those high in calories, saturated fats and sugars conversely leads to negative consequences to brain functioning. Together, these results suggest that dietary factors should be a major consideration when attempting to maximize neuroplasticity induced by training or learning. An individual’s dietary habits will also be valuable information for researchers trying to understand individual differences in responses to the same intervention attempting to induce neuroplasticity, such as exercise or non-invasive brain stimulation.

### Combination of Diet and Exercise

Evidence suggests that the combination of diet and exercise provides synergistic benefits (Kishi and Sunagawa, 2012; Murphy et al., 2014; Hutton et al., 2015). Exercise combined with caloric restriction enhanced BDNF levels in mice more than fasting or exercise alone (Stranahan et al., 2009). Supplementation of omega-3 fatty acids such as DHA increased rat hippocampal BDNF levels, and this effect is increased when applied concurrently with exercise (Wu et al., 2008). Further, axonal growth and synaptic function were enhanced by a 1.25% DHA diet and exercise more than DHA or exercise alone (Chytrova et al., 2010). A study comparing the effects of a 6-week polyphenol-rich diet alone to a regimen combining this diet with aerobic exercise (treadmill running) on spatial memory in mice found that the combination of diet and exercise evoked greater enhancements in spatial memory, and these changes were associated with increased angiogenesis and neuronal spine density in the hippocampus (van Praag et al., 2007). Further, there was an upregulation of genes associated with learning, alongside decreases in genes associated with neurodegeneration in the neurons of the hippocampus (van Praag et al., 2007).

Long-term adherence to both an aerobic exercise and healthy diet regimen such as the Mediterranean Diet (Hardman et al., 2020) or minimum consumption of > 400 g/day of fruits/vegetables and > 2 servings of fish per week led to significant improvements in cognition within middle-aged and older adults (Komulainen et al., 2021). This is particularly important, because these diets alone were not reported to induce long-term cognitive benefits (Hardman et al., 2020; Komulainen et al., 2021). Further, another study recruited 148 active Air Force Airmen and placed them on a 12-week randomized controlled trial. The intervention was characterized by combining aerobic and strength training with a novel nutritional supplement [comprised of β-hydroxy β-methylbutyrate (HMB), lutein, phospholipids, DHA and selected micronutrients including B12 and folic acid] which led to greater improvements in working memory and reaction time compared to aerobic and strength training alone (Zwilling et al., 2020). This evidence suggests that exercise-induced neuroplasticity may be facilitated by modifications in diet. However, these results may have limited generalizability since this study was done in healthy, young, highly trained military personnel only. Therefore, even though the study has uncovered a plausible link, further research to quantify the brain changes via neuroimaging must be done to uncover the effect of these multimodal interventions. Furthermore, future directions would require an individualized dietary-exercise regime as individual physiological variability is rather robust.

## Sleep

Sleep is an important factor implicated in brain plasticity (Frank, 2015), recovery from brain injury (Jasey and Ward, 2019), as well as cognitive functioning, learning and memory (Ellenbogen, 2005). Neuroplasticity is facilitated by the adequate delivery of cerebral blood flow to supply active neurons with oxygen while simultaneously removing waste products. There are dynamic changes in cerebral blood flow during the sleep-wake cycle, and these involuntary patterns may have an influence on the passive processes of neuroplasticity that occur during sleep (Elvsåshagen et al., 2019). Sleep quantity also influences active processes of neuroplasticity, as evidenced by the influence of sleep deprivation on task-related cerebral blood flow and cognitive functioning. Twenty-four hours of sleep deprivation resulted in impaired attention and reaction time, as well as decreased cerebral blood flow from the right middle cerebral artery during a finger tapping task in young healthy males (Csipo et al., 2021). Similarly, cerebral blood flow has been shown to be reduced in the cerebellum, cuneus and fusiform gyrus of the right hemisphere, along with increased cerebral blood flow in the inferior occipital gyrus in the opposite hemisphere in night shift workers compared to daytime workers (Park et al., 2019). This study also noted that these decreases were significantly correlated with self-reported severity of depression and insomnia. Together, these findings suggest that decreased cerebral blood flow induced by poor sleep can have a negative influence on cognitive functioning and processes of neuroplasticity. Sleep also influences the regulation of several important neurotrophic factors that could mediate the influence of sleep on neuroplasticity. For example, sleep regulates the growth hormone (GH)/IGF-1 axis such that IGF-1 levels are decreased in the context of “sleep loss” and increased in situations of “sleep extension” (Chennaoui et al., 2020). Similarly, sleep disturbances over a long-term period leads to decreased BDNF levels, whereas acute sleep deprivation causes an increase in BDNF levels as part of the physiological stress response in humans (Schmitt et al., 2016).

Sleep deprivation is known to alter brain excitability and intracortical inhibition, which may consequently diminish the potential for neuroplasticity (Kreuzer et al., 2011). Specifically, studies investigating the influence of sleep deprivation on TMS measures have consistently reported a decrease in SICI and CSP in comparison to a night of normal sleep (Scalise et al., 2006; Kreuzer et al., 2011; Placidi et al., 2013). Likewise, conditions including insomnia, restless leg syndrome or sleep apnea (which are characterized by a reduced quality and/or quantity of sleep) have abnormal single- and paired-pulse TMS measures of cortical excitability and inhibition compared to control groups as reviewed previously (Lanza et al., 2015). M1 excitability is decreased in sleep apnea and increased in RLS. Other conditions such as insomnia and sleep deprivation present imbalanced inhibitory/excitatory intracortical circuitry resulting in heightened intracortical inhibition (Lanza et al., 2015). Further, individuals with RLS and sleep apnea have demonstrated irregular responses to TMS-induced plasticity. Repetitive TMS (rTMS) is a plasticity-inducing TMS paradigm consisting of a rapid (5–10 Hz) succession of pulses delivered to the cortex which induces lasting changes in brain excitability (Fitzgerald et al., 2006). A 10 Hz rTMS over M1 increased MEP amplitude in healthy controls, but not in untreated patients with severe obstructive sleep apnea (Das et al., 2013). Continuous theta-burst stimulation (cTBS) is form of rTMS involving clusters of three pulses delivered in rapid succession, which is believed to induce LTD and suppress cortical excitability (Huang et al., 2005). When performed in patients with sleep apnea, cTBS at 50 Hz over the left M1 did not decrease M1 excitability unlike the decrease seen in healthy controls (Opie et al., 2013). Finally, paired-associative stimulation (PAS) is a paradigm involving the repeated pairing TMS and peripheral electrical nerve stimulation which can strengthen corticospinal excitability (Alder et al., 2019). Delivery of a 90 pulse PAS protocol increased corticospinal excitability in healthy controls but not patients with RLS. However, the propensity for associative plasticity was restored in patients following 4 weeks of dopaminergic treatment (Rizzo et al., 2009). These disrupted responses to plasticity-inducing paradigms in individual’s with sleep disorders suggest that normal processes of neuroplasticity are dysregulated by a reduction in sleep. These results highlight how the proper quantity and quality of sleep is essential to promote neuroplasticity in the brain. The amount of sleep that an individual should get every night depends on their age and other health factors. For further information on sleep recommendations, see Ohayon et al. (2017).

Sleep can largely be divided into non-rapid eye movement (NREM) and rapid eye movement (REM) stages, with the NREM stages further divided into NREM 1, 2, 3, and 4. During normal human sleep, individuals cycle through these stages every 90 min, with the ratio of REM/NREM shifting from largely NREM 3 and 4 in the first half, to primarily NREM 1, 2 and REM sleep later in the night (Walker, 2009). Each of these stages is characterized by distinct brain activity and serves a unique role in the restoration and maintenance of the body during sleep (Moser et al., 2009). Due to these differences, certain stages of sleep may have a more pronounced effect on neuroplasticity than others. For example, REM sleep is known to actively involve processes of neuroplasticity in the hippocampus during the night (Frank, 2015). These changes are thought to have implications with consolidation of learning and memory (Maquet, 2001). When comparing NREM to REM sleep deprivation, REM sleep deprivation resulted in a reduction of CSP and SICI, while NREM sleep deprivation did not change these measures (Placidi et al., 2013). These findings suggest that REM sleep may have significant influences on processes of neuroplasticity during waking hours. In contrast, another study done in rats found that sleep continuity, but not the amount of REM sleep, was critical for learning and memory on a novel object recognition task (Rolls et al., 2011). However, this discrepancy may be due to the difference in species being investigated in these two studies. To test this theory, future research should develop more investigation into the role of REM sleep on neuroplasticity in the human brain using different neuroimaging and neurophysiological techniques.

There is evidence for the effect of sleep on memory processing at the molecular, neuronal, systems and behavioral level (Maquet, 2001). However, the neurophysiological mechanisms underlying these effects are not yet fully understood (Walker, 2009). The inconsistent results mentioned in this review demonstrate the need for more studies to directly compare the effect of REM to NREM sleep deprivation on memory and other neurophysiological measures of plasticity. It has been shown that memory consolidation and synaptic remodeling processes occur actively while learning (Mansvelder et al., 2019). Interestingly though, these processes have also been shown to continue during sleep due to mechanisms related to post-learning plasticity (Stee and Peigneux, 2021). These findings highlight the importance of sleep quality before and after learning or training when attempting to promote neuroplasticity.

In conclusion, sleep is a complex biological process which has a multitude of effects on neuroplastic processes in the brain, both during sleep and while awake. Our findings here would suggest that getting the adequate amount of sleep based on recommended guidelines (Ohayon et al., 2017) may increase the propensity for neuroplasticity in the brain. Alternatively, sleep deprivation, reduced REM sleep duration, or the presence of a sleep disorder may result in disrupted neuroplastic processes which can have a negative influence on memory consolidation during sleep, as well as motor control and cognition during waking hours. These results highlight the importance of controlling for an individual’s sleep status in studies measuring neuroplasticity, and of promoting good sleeping habits to allow for optimal performance in cognitive or physical tasks.

### Combination of Sleep and Exercise

Few studies have investigated the combined influences of exercise and sleep on neuroplasticity. One recent study found that higher levels of daily physical activity at the expense of sedentary behaviors and sleep are associated with greater cortical neuroplasticity (Smith et al., 2021). Interestingly, it was shown that the increases in neuroplasticity were equal regardless of whether physical activity replaces time spent sedentary or sleeping (Smith et al., 2021). However, evidence showing disrupted neuroplasticity due to poor sleep discussed in the previous section of this review would suggest that the most beneficial decision for neuroplasticity would be to replace sedentary activity with physical activity rather than sacrifice your sleep. Sleep quality has been identified as a mediator of the relationship between physical activity and neurocognitive function along with stress, mood and pain (Stillman et al., 2016). Sleep efficiency (total amount of time spent sleeping in bed) also mediated the relationship between physical activity and multiple measures of cognitive performance including working memory, recall and verbal skills in younger and older adults (Wilckens et al., 2018). Exercise, sleep and diet are all important factors contributing to the neural pathways involved in major depression which is characterized by disrupted neural processes related to plasticity inducing neurotransmitter imbalances, HPA-axis disturbances, oxidative stress, mitochondrial disturbances and inflammation (Lopresti et al., 2013). Exercise and sleep are also primary factors that influence the maximization of recovery from brain injury and neuroplasticity (Jasey and Ward, 2019).

Higher physical activity levels are associated with better sleep, so the interaction between these two factors may be particularly important. For example, in a 16-week randomized control trial to determine the effects of exercise training on sleep in healthy older adults, individuals in the exercise group displayed significant improvements in the global sleep score on the Pittsburgh Sleep Quality Index (PSQI) compared to a non-exercising control group (King et al., 1997). In another longitudinal study of older adults, better sleep quality at baseline predicted higher levels of physical activity at the follow up measurement, regardless of prior physical activity (Holfeld and Ruthig, 2014). Further, baseline physical activity levels did not predict sleep quality at the follow up (Holfeld and Ruthig, 2014). Together, these findings suggest a bidirectional relationship between physical activity and sleep quality, though further investigation is necessary to determine the underlying mechanisms mediating this relationship. Since these studies were both done in healthy older adults, future work should investigate this relationship in different age groups and demographic populations to ensure that these findings are not limited to this group only.

Several animal model studies have shown that engaging in exercise may be able to prevent sleep-deprivation related reductions in neuroplasticity in mice and rats. Seventy-two hours of sleep deprivation leads to aberrant signaling in the hippocampus and impaired short-term memory performance in mice when compared to a sedentary control group, but these changes were prevented by aerobic exercise prior to the sleep deprivation (Tuan et al., 2021). Authors suggested that aerobic exercise had a neuroprotective effect on microglia-mediated synaptic pruning that occurs in sleep-deprived brains and can be used as a method to cope with sleep insufficiency (Tuan et al., 2021). Two other studies exploring this relationship in rats have found that regular treadmill running prevented sleep-deprivation related disruptions in synaptic plasticity and neuronal signaling (Zagaar et al., 2013) as well as late-phase LTP in the dentate gyrus (Zagaar et al., 2016). Finally, 4 weeks of voluntary aerobic exercise in female rats prevented sleep deprivation related impairments in early-phase LTP in the hippocampus along with reductions in behavioral assessments of learning and memory (Rajizadeh et al., 2020). Collectively, these findings support the existence of a complex interaction between exercise and sleep that is mediated by several neurophysiological mechanisms not yet fully understood. Further investigation is required to comprehensively understand the protective effects of exercise on the negative health consequences of sleep-deprivation, as well as the ideal combination of sleep and exercise to prime neuroplasticity.

### Combination of Sleep and Diet

Previous research studies have suggested a bidirectional relationship between diet and sleep quality that is mediated through mechanisms related to neuroinflammation, neurogenesis, and neuroplasticity (Godos et al., 2020a,b, 2021). Several studies have investigated the influence of diet on sleep in healthy and clinical populations. Adherence to a Mediterranean diet over 2 years was associated with lower risk of poor sleep quality and duration compared to those with lower adherence (Campanini et al., 2017). Similarly, another study revealed that adherence to a Mediterranean diet was associated with better sleep quality (Godos et al., 2019). The association between adherence to this diet and sleep quality may be indirectly mediated by reduced weight and improved body composition (Godos et al., 2019). Related findings were reported in a study that examined the effects of a ketogenic diet in adolescents with morbid obesity. The dietary intervention induced weight loss that consequently led to increases in REM sleep and decreases in NREM sleep toward normal levels. This was suggested to be due to less obstructive blockage of the airways during sleep (Willi et al., 1998).

Another study evaluated the antiepileptic properties of the ketogenic diet by exploring it’s effect on sleep in children with epilepsy. Adherence to the ketogenic diet increased REM sleep duration and improved sleep quality, which contributed to an increased in quality of life (Hallböök et al., 2007). Findings from this study are limited by it’s small sample of 18 participants all younger than 18 years of age, so more work must be done to confirm these findings and extend them into other age groups. In another study, improvements in several health measures including sleep quality and daytime sleepiness resulted in improved quality of life following ketogenic dietary intervention in patients with obesity (Castro et al., 2018). Although limited by a small sample size, these promising results may also help contribute to an increasing use of the ketogenic diet as a form of therapy in the upcoming years.

Results from studies exploring the combination of sleep and diet demonstrate a clear interplay between these lifestyle factors. In summary, maintaining or introducing a healthy diet will result in positive outcomes in the brain which have beneficial effects on sleep quality. This interplay between factors suggests that the proper combination of diet and sleep may result in the optimization of the brains ability to undergo neuroplasticity. Despite the abundance of evidence highlighting the relationship between sleep, diet, and brain function, no study has been done to directly understand how different combinations of sleep quality or quantity and dietary interventions effect the propensity for neuroplasticity in response to exercise or non-invasive plasticity-inducing protocols like rTMS or PAS.

## Limitations and Future Directions

This manuscript does not provide an exhaustive review on the literature related to exercise, diet, sleep and neuroplasticity. Instead, the intention is to provide a comprehensive understanding of each of these topics and their combinations using publications that have directed these fields of research. Importantly, this review serves as an opportunity to identify gaps in knowledge for which future research directions will pursue.

Future research studies are encouraged to compare the neurophysiological responses to aerobic, resistance and combined training in the context of acute and long-term exercise programs in both healthy and clinical populations in order to translate findings to neurorehabilitation. The intensity of the exercise protocol and fitness level of individuals involved are particularly important factors that must be considered when designing these studies. Further, researchers might consider assessing responses in the resting and active muscle states to fully elucidate the neuroplastic adaptations induced by exercise training paradigms. These studies will provide invaluable information regarding the differences in neurophysiological adaptations to these two types of exercise and how a combination of the two may provide additional neural benefits.

However, there are also alternative forms of exercise that exist such as balance training, flexibility training, and other complex or coordinative exercises. These types of exercise were not discussed in this review because there is a limited amount of research on these topics, and the purpose of this review was to discuss the most common types of exercise used most frequently. Yet, evidence suggests that these types of exercise also possess the potential to influence the structure and function of the brain in healthy individuals (Rogge et al., 2018) as well as in special populations such as individuals with Parkinson’s Disease (Sehm et al., 2014). Therefore, future work should also investigate how these alternative forms of exercise influence measures of neuroplasticity in isolation, and in combination with aerobic and resistance exercise.

In relation to diet, it is suggested that future research explores popular and emerging diets from a neurophysiological perspective to improve our understanding of the potential benefits they offer in conjunction with exercise training and sleep quality. Also, dietary supplements with reported benefits to learning, memory or brain function can be assessed using neurophysiological measurements such as TMS or changes in biomarkers like BDNF or IGF-1. Doing so will allow for researchers to determine if there is any evidence of neuroplasticity following consumption, or if regular/prolonged consumption leads to lasting changes in brain excitability.

There is a need for further investigation into the influence of the stages of sleep on the neuroplastic responses to interventions such as exercise or rTMS by assessing the effect of total or partial sleep deprivation on neurophysiological measures of neuroplasticity, similar to the work done by Kreuzer et al. (2011). This work will be important to understand the complex relationship between REM sleep and neuroplasticity, as well as to elucidate the role of NREM sleep in these processes.

Researchers measuring neuroplasticity in future studies are strongly encouraged to incorporate assessments of physical activity, sleep quality and eating/dietary habits at baseline and throughout the duration of longitudinal studies. This practice can be used to evaluate the influence of these factors as mediators of the relationship between the intervention and neuroplasticity in a study. Examples of commonly used tools that can evaluate these factors include the PSQI for sleep, International Physical Activity Questionnaire (IPAQ) for exercise, and food logs for diet. Finally, researchers should continue to identify other lifestyle factors that also may influence neuroplasticity. Some examples of other factors to explore include mood, motivation, mindfulness, stress, socioeconomic status, intelligence, education level, and substance use. In addition, it is recommended that experimenters systematically investigate the interplay of exercise, diet and sleep in relation to neuroplasticity in a single study to converge on the optimal balance of these factors necessary to promote neuroplasticity.

## Conclusion

Neuroplasticity underlies the basis of learning as well as recovery from brain injury and neurological impairment, making it an essential component of the research and neurorehabilitation efforts. We reviewed evidence from molecular, systems and behavioral neuroscience from human and animal models to summarize the influence of exercise, diet, and sleep patterns on neuroplasticity. Studies investigating how the combination of these factors affects neuroplasticity were also discussed, highlighting the importance of a holistic approach when aiming to induce neuroplasticity. Nevertheless, it is important to note that this paper does not provide an exhaustive list, and researchers should continue to identify the role of other potential lifestyle factors to expand our current understanding of how they influence the neurophysiological mechanisms underpinning neuroplasticity. Findings from such studies will continue provide valuable insight into how future research should control for these fundamental lifestyle factors in their studies. Examples of methods to control for these factors includes the screening process, inclusion/exclusion criteria, baseline and outcome measures recorded, and forms of analysis adopted. We also offered several suggestions for future studies to address some important gaps in the literature that will be vital in progressing toward a more efficient induction of neuroplasticity for research, learning, fitness, and rehabilitative purposes.

## Author Contributions

JP, CT, and KR: writing – original draft and writing – review and editing. RR and SF: writing – review and editing. AN: funding acquisition and writing – review and editing. All authors contributed to manuscript revision, read, and approved the submitted version.

## Conflict of Interest

The authors declare that the research was conducted in the absence of any commercial or financial relationships that could be construed as a potential conflict of interest.

## Publisher’s Note

All claims expressed in this article are solely those of the authors and do not necessarily represent those of their affiliated organizations, or those of the publisher, the editors and the reviewers. Any product that may be evaluated in this article, or claim that may be made by its manufacturer, is not guaranteed or endorsed by the publisher.

## Abbreviations

BDNF: brain-derived neurotrophic factor
CSP: contralateral silent period
cTBS: continuous theta-burst stimulation
DHA: Docosahexaenoic acid
EEG: electroencephalography
ERP: event-related potential
fMRI: functional magnetic resonance imaging
ICF: intracortical facilitation
IGF-1: insulin-like growth factor 1
IPAQ: international physical activity questionnaire
LICI: long-interval intracortical inhibition
LTD: long-term depression
LTP: long-term potentiation
M1: primary motor cortex
MEP: motor-evoked potential
MMSE: mini mental status examination
MoCA: Montreal Cognitive Assessment
NREM: non-rapid eye movement
PAS: paired-associative stimulation
PKC: protein kinase C
PSQI: Pittsburgh Sleep Quality Index
REM: rapid eye movement
RMT: resting motor threshold
rTMS: repetitive TMS
SAI: short-latency afferent inhibition
SICI: short-interval intracortical inhibition
SIS: Stroke Impact Scale
VEGF: vascular endothelial growth factor.
